# *Aspergillus flavus* Exploits Maize Kernels Using an “Orphan” Secondary Metabolite Cluster

**DOI:** 10.3390/ijms21218213

**Published:** 2020-11-03

**Authors:** Ludovica Antiga, Sonia Roberta La Starza, Cecilia Miccoli, Simone D’Angeli, Valeria Scala, Marco Zaccaria, Xiaomei Shu, Gregory Obrian, Marzia Beccaccioli, Gary A. Payne, Massimo Reverberi

**Affiliations:** 1 Department of Environmental Biology, Sapienza University of Rome, P.le Aldo Moro 5, 00185 Roma, Italy; ludovicaantiga@gmail.com (L.A.); lastarza.soniaroberta@gmail.com (S.R.L.S.); simone.dangeli@uniroma1.it (S.D.); massimo.reverberi@uniroma1.it (M.R.); 2 CREA-OFA, Via di Fioranello 52, 00134 Rome, Italy; cecilia.miccoli@crea.gov.it; 3 CREA-DC, via C.G. Bertero 22, 00156 Roma, Italy; valeria.scala@crea.gov.it; 4 Department of Biology, Boston College, 140 Commonwealth avenue, Chestnut Hill, MA 02467, USA; zaccarim@bc.edu; 5 Center for Biotechnology and Genomics, Texas Tech University, 2500 Broadway, Lubbock, TX 79410, USA; shuxiaomei@gmail.com; 6 Department of Plant Pathology, North Carolina State University, Raleigh, CA 27607, USA; gregoryobrian@gmail.com (G.O.); gary.a.payne@gmail.com (G.A.P.)

**Keywords:** *Aspergillus flavus*, maize kernel, salicylate hydroxylase, npp1, effectors, quercetin, histology

## Abstract

*Aspergillus flavus* is a saprophytic cosmopolitan fungus, capable of infecting crops both pre- and post-harvest and exploiting different secondary metabolites, including aflatoxins. Aflatoxins are known carcinogens to animals and humans, but display no clear effect in host plants such as maize. In a previous study, we mined the genome of *A. flavus* to identify secondary metabolite clusters putatively involving the pathogenesis process in maize. We now focus on cluster 32, encoding for fungal effectors such as salicylate hydroxylase (*SalOH*), and necrosis- and ethylene-inducing proteins (npp1 domain protein) whose expression is triggered upon kernel contact. In order to understand the role of this genetic cluster in maize kernel infection, mutants of *A. flavus*, impaired or enhanced in specific functions (e.g., cluster 32 overexpression), were studied for their ability to cause disease. Within this frame, we conducted histological and histochemical experiments to verify the expression of specific genes within the cluster (e.g., *SalOH*, *npp1*), the production of salicylate, and the presence of its dehydroxylated form. Results suggest that the initial phase of fungal infection (2 days) of the living tissues of maize kernels (e.g., aleuron) coincides with a significant increase of fungal effectors such as *SalOH* and *Npp1* that appear to be instrumental in eluding host defences and colonising the starch-enriched tissues, and therefore suggest a role of cluster 32 to the onset of infection.

## 1. Introduction

*Aspergillus* is one of the best-described fungal *genera*, mainly because of its negative impact on economy and agriculture through production of highly toxic secondary metabolites [1]. *Aspergillus flavus* is studied for its role in producing aflatoxins (AFs), the most carcinogenic natural substances currently detected in food. *A. flavus* is more virulent than most *Aspergillus* species; furthermore, *A. flavus* causes a broad spectrum of disease in humans ranging from cutaneous to central nervous system infections [2]. 

*A. flavus* is a nutrient recycler abundant in the soil [2], which often opportunistically infects staple crops, such as maize and leads to aflatoxin contamination [3,4,5,6]. Maize (*Zea mays* L.) is one of the major crops susceptible to *A. flavus* infection and subsequent aflatoxin contamination [7]. One of the earliest reports of infection on maize by *Aspergillus* was in Texas in 1920. Aflatoxins contamination of maize was thought to be a post-harvest consequence of improper storage. However, research has indicated that *Aspergillus* infection may occur pre-harvest [8]. In the last years, contamination levels in Europe have exceeded the threshold allowed by EFSA [9] for commerce. Improvement strategies have thus been necessary, e.g., dilution of the contaminated commodities with safer grain, or chemical treatment to destroy or inactivate aflatoxins [10,11]. Maize kernels are generally susceptible to infection by *A. flavus*, yet aflatoxin production in corn is extremely variable due, in part, to its sporadic occurrence among ears and kernels. The fungal structures associated with aflatoxins (e.g., conidial heads and sclerotia) likely exhibit a similar sporadic distribution pattern between maize ear and kernel [3]. Over recent years, in the U.S., *A. flavus* infection caused 100s of millions USD of yearly losses due to aflatoxin contaminated crops [12]. The studies on maize-*A. flavus* interaction have revealed that additive and dominant gene actions are important for resistance to aflatoxin production. Diallel mating designs were employed to study the inheritance of resistance to both *A. flavus* ear rot and aflatoxin accumulation. These studies have reported that general combining ability had a greater effect on the aflatoxin resistance in maize than specific combining ability, suggesting that additive gene effect is more important than dominant gene effect. Transgenic approaches have been attempted to manage *A. flavus* infection and improve food safety [13].

Secondary metabolites (SM) play an important role in fungal ecology as fitness factors, but they are, by definition, not essential for growth or survival. SMs are structurally heterogeneous and their coding sequences are organized in clusters, each cluster containing enzymatic genes and often transcriptional factors [1,14]. In *A. flavus*, SMURF (Secondary Metabolite Unknown Regions Finder- available at http://www.jcvi.org/smurf) predicted at least 56 different clusters for production of secondary metabolites [15], which can be divided in four clades based on hierarchical clustering. Following this from the division in clades, and the work by Georgianna et al. [16] in which connections between ecological conditions and gene expression were first shown, Reverberi et al. [17] examined genotypic and phenotypic changes in *A. flavus* during its growth and invasion of maize kernels: out of 56 secondary metabolites clusters, 24 were differentially expressed. Among the secondary metabolites up-regulated during maize kernel infection, cluster 32 emerged as the most interesting since it had not been previously implicated in pathogenicity. At least two genes within cluster 32, *DMATS* and *GGPS*, are implied in the biosynthesis of aflatrem, a tremorgenic mycotoxin causing neurological disorders [17,18,19]. Intriguingly, in this study we found that a set of genes within cluster 32 co-expressed with the necrosis- and ethylene-inducing peptide (npp1), which belongs to the Nep1-Like Proteins (NLPs), a family of non-host-specific elicitors causing necrosis and activating defence responses in dicotyledonous plants [20,21]. NLPs are small phytotoxic conserved proteins able to induce hypersensitive response (HR)-like cell death, reactive oxygen species (ROS) and ethylene production. Recent structural analyses indicate that NLPs are similar to virulence- promoting cytolytic toxins and act by interfering with integrity of the plasma membrane; the activation of host defences starts with the disruption of plasma membrane [22]. 

Current approaches are insufficient to control pre-harvest colonisation of crops by *A. flavus* and subsequent AF contamination. Genetic strategies could enable the development of new methodologies to control the pathogen and, consequently, AF biosynthesis. To this end, it is important to gain insight into the genetic regulatory pathways that control *A. flavus* morphogenesis and toxin biosynthesis [23].

We focused our attention on the transcriptional regulation of cluster 32, which we define a “orphan” cluster since its function is hitherto unknown. For the sake of clarity, we aim at defining cluster 32 within this work. Previous bioinformatic analysis indicated that a putative actor in this process could be the *AFLA_096370* gene encoding for a DNA-binding zinc protein, *Zn_2_Cys_6_*, transcriptional factor. The fungal C_6_ zinc-finger cluster proteins are normally associated with the regulation of different functions including carbon and nitrogen utilization, production of secondary metabolites, and asexual and sexual development [24]. In this study, we analysed the behaviour of an *A. flavus* strain overexpressing *AFLA_096370* as well as both knocked-out and over-expressing NepA, a necrosis and ethylene-inducing peptide, to assess the role of cluster 32 in the pathogenic process of *A. flavus* on maize kernels.

## 2. Results & Discussions

### 2.1. Phenotypic Characterization of A. flavus Mutant Strain

Our first approach was to characterize the morphology of the strains by visual observation. AF3357 (wild type) and AFC-1, a mutant (−*pyrG*, −*argD*) auxotroph for uracil and arginine, had similar total growth, but AF3357 grew faster and produced more conidia. *Zn_2_Cys_6_-OE-GFP* mutant, which overexpresses the transcription factor, showed hampered growth but strong conidiation. The knockout for the transcription factor, *Zn_2_Cys_6_∆*, displayed faster growth but weaker conidiation. All the evaluations were made 7 days after plating (Figure S1). 

### 2.2. Colonisation of Maize Kernels by A. flavus AF3357, AFC-1 and Zn_2_Cys_6_-OE-GFP

**Figure 1:**
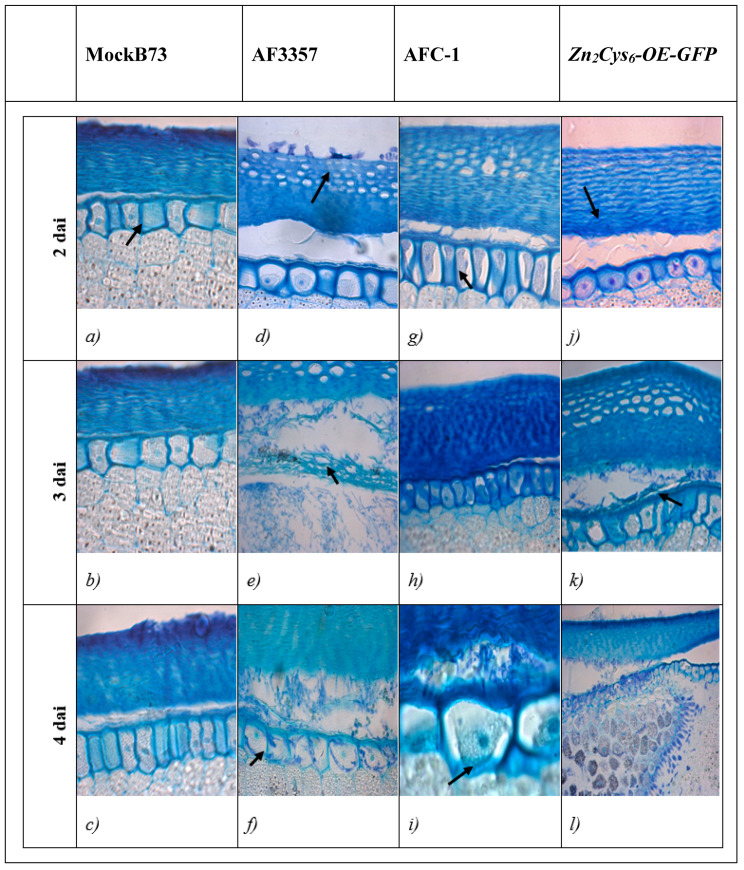
Histological assays showing the progression of the infection by different strains at 2, 3 and 4 dai. MockB73 (**a**–**c**) tissues were not infected at any time point. Wild-type AF3357 (**d**–**f**) spores are present just outside the kernel at 2 dai (**d**), and at 4 dai the fungus had directly invaded the aleuronic cells as shown by the arrow (**f**). Starting at 2 dai, the AFC-1 mutant (**g**–**i**) experienced visible vacuolisation of aleuronic cells (arrows in **g**) as symptoms of PCD onset, progressing to 4 dai with ongoing infection (**i**). The transcription factor overexpressing mutant, *Zn_2_Cys_6_-OE-GFP* (**j**–**l**), as a consequence of fungal invasion caused detachment of the periderm from the aleuronic layer (arrow in **j**), then proceeds just outside the aleuronic layer (arrow in **k**) until the kernel is collapsed (**l**). Magnification 400×.

Colonisation of maize kernels was observed for several strains by in vitro infection assay (Figure S2); histological sections were observed with an optical microscope. At 2 days after infection (dai), AF3357 had completely colonised the pericarp and produced a visible mycelial mat (Figure 1b); at 3 dai, conidia were present in the aleurone layer and within the pericarp (Figure 1f); and at 4 dai, every cell of the aleuronic layer had been invaded completely (Figure 1j). Regarding AFC-1 and *Zn_2_Cys_6_-OE-GFP* strains, no sign of fungal contamination appeared (conidia or mycelial mat) within the kernels at 2 dai (Figure 1c,d). In the sole *Zn_2_Cys_6_-OE-GFP* mutant, the pericarp and the aleuronic layer, but not the embryo, were invaded at 3 dai (Figure 1h); at 4 dai, AFC-1 behaved similarly to *Zn_2_Cys_6_-OE-GFP* in that a dense mycelial mat was visible between the pericarp and the aleuronic layer (Figure 1k). Notwithstanding the difference in the rate of invasion among the three strains, the aleuronic layer might represent a barrier to early-stage infection (Figure 1f,j,l), but as the disease progresses the kernel eventually collapse (at 7 dai, data not shown due to kernels high deterioration). The *Zn_2_Cys_6_∆* strain showed low potential for maize kernels invasion, because at 7 dai, no signs of infection could be observed except for a minimal growth on the external surface of the kernel (Figure S3). 

**Figure 2:**
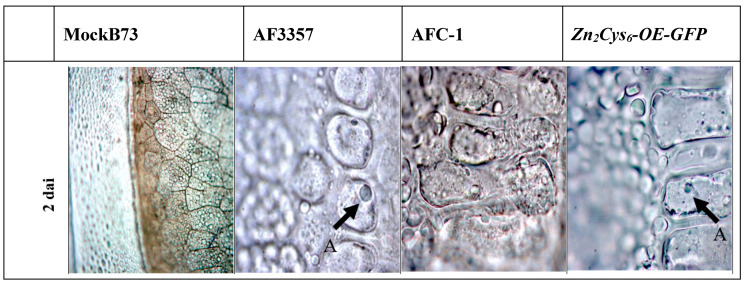
TUNEL assay at 2 dai in all the studied strains: MockB73, AF3357 (wild type); AFC-1 (−*pyrG*, −*argD* mutant); *Zn_2_Cys_6_-OE-GFP* (overexpressing the transcription factor). In all three cases, the cells belong to the aleuronic layer. This assay shows apoptotic (A) nuclei in blue indicated by the arrows. Magnification 1000×.

Post-infection programmed cell death (PCD) occurred in the aleuronic layer at different dai among the strains (AF3357 < *Zn_2_Cys_6_-OE-GFP* < AFC-1), (Figure 1f,h,k). The timing of PCD occurrences is better highlighted with the TUNEL assay (Figure 2) where blue nuclei, indicative of PCD onset, are brighter in AF3357-infected kernels than in the other samples. We also report decreased PCD in *Zn_2_Cys_6_∆-* infected kernels through TUNEL assay (Figure S4). 

Comparison with viable aleuronic cells revealed vacuolization in the PCD-affected cells starting from 2 dai (Figure 1b–d,f–h,j–l); moreover, the protein storage vacuoles disappeared in the PCD-affected cells, whereas the viable cells were filled with these vacuoles (arrow in Figure 1a). Vacuoles were similar to the vesicles preceding PCD in the aerenchyma formation in maize and may be interpreted as lytic vacuoles [25].

### 2.3. Expression of Genes Related to the Pathogenesis 

Salicylic acid (SA) signalling pathways play an important role in plant defence [26]. To counteract the effect of SA synthesis by the plant, some pathogens (e.g., hemi-biotrophic ones) can degrade it. These pathogens, which include a number of fungal species, can convert SA to catechol and/or gentisate, directly or through intermediates [27]. This reaction is accomplished by a specific enzyme known as salicylate hydroxylase. In *Arabidopsis*, SA induction-deficient mutants, inactivated for the isochorismate synthase gene, fail to synthesize SA in response to pathogen infection, resulting in enhanced susceptibility to bacterial and fungal pathogens [28]. Thus, hemi-biotrophic pathogens often jeopardize the SA pathway to delay PCD, especially during the biotrophic stage. Indeed, in the later, necrotrophic stage, these pathogens prompt cell death [29]. Regarding this aspect, Npp1 is a necrosis- and ethylene-inducing peptide domain present in all NLPs. It has been proposed to have a dual function in plant–pathogen interactions, acting both as a trigger of immune response and as a toxin-like virulence factor [30]. NLPs are relatively small proteins of about 24 kDa that exhibit a high degree of similarity at amino acid sequence level, including the presence of two highly conserved cysteine residues that form an intramolecular disulfide bridge essential for NLP activities, and also a central hepta-peptide motif “GHRHDWE” that is part of the negatively charged cavity exposed at the protein surface. Both are necessary for plasma membrane permeabilization and cytolysis in plant cells [31]. Plants produce phenolic compounds for pigmentation, growth, and reproduction even under stressors such as wounding, drought, and pathogen attack [32]. The latter can deeply affect the synthesis and accumulation of phenolics by rutin and quercetin, amongst the main phenolic antioxidant compounds in plants [33]. It is largely accepted that oxidative stress plays an important role as an early factor in establishing the fate of pathogen/plant interactions. In relation to this, host cells have based an important part of their defence toolkit on antioxidant activities, which are triggered soon after pathogen infection [34]. It appears crucial for the fungal pathogen to disable this type of defence to achieve tissue colonisation. 

Following up on our previous work in which we reported a correlation between cluster 32 and the pathogenicity process of *A. flavus* in maize [17], we focused our attention in this study on three genes inside this cluster: salicylate hydroxylase (*SalOH*), the gene responsible for necrosis- and ethylene-inducing protein (*npp1*), and quercetin dehydrogenase (*AFLA_096260*).

**Figure 3:**
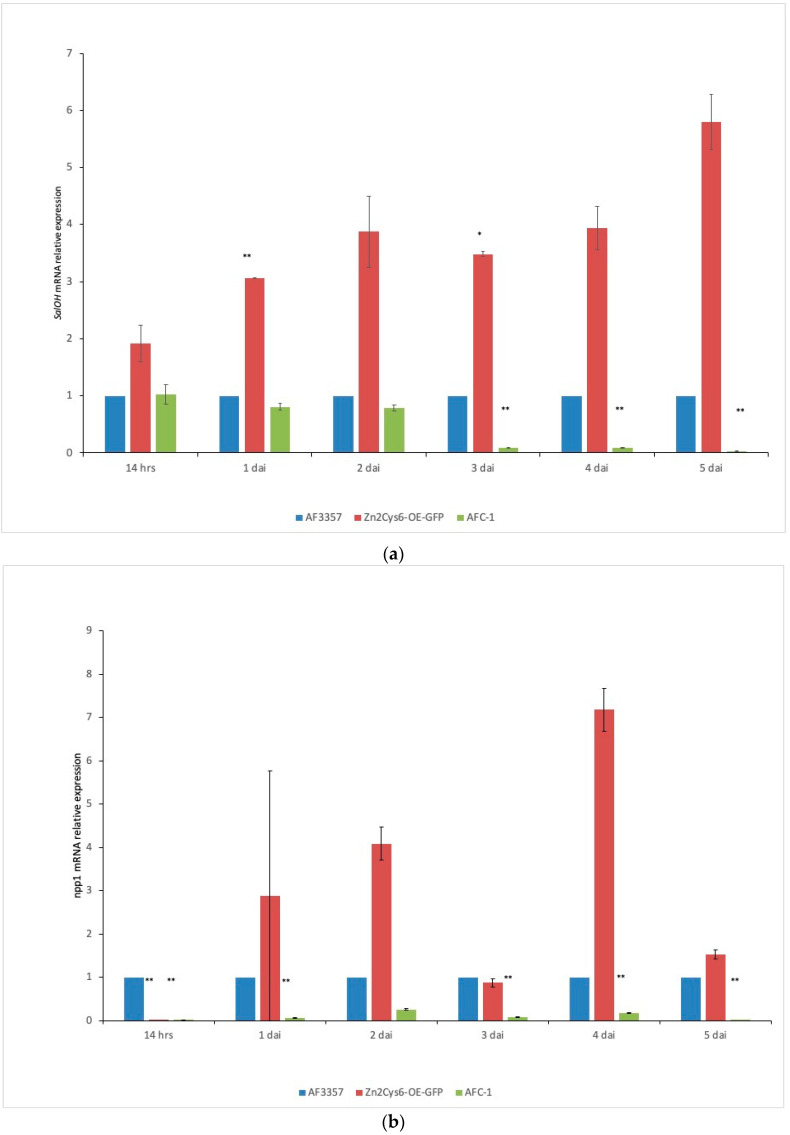

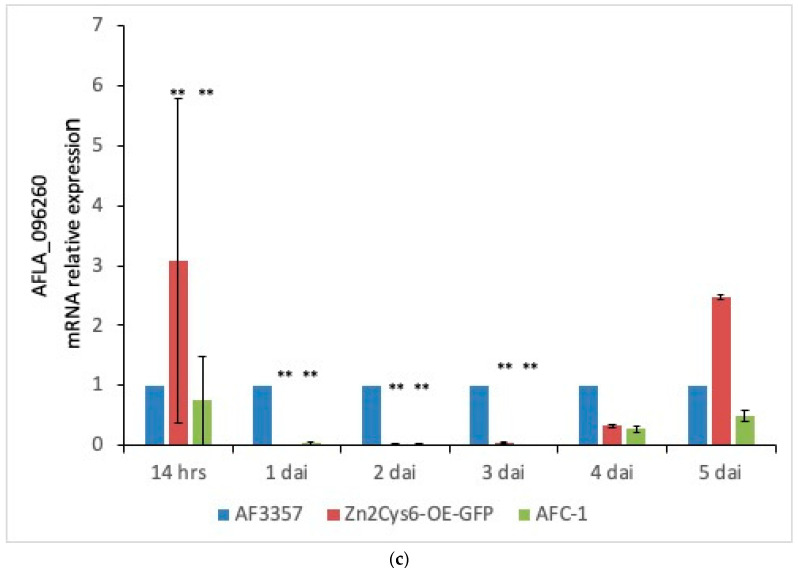
Relative expression of transcriptional levels for cluster 32 genes *SalOH* (**a**), *npp1* (**b**) and AFLA_096260 (**c**) in fungal strains AF3357, *Zn_2_Cys_6_-OE-GFP* and AFC-1 from 14 hrs to 5 dai. Mean values and standard deviations were used to perform *t*-tests comparing *Zn_2_Cys_6_-OE-GFP* or AFC-1 to AF3357 (* *p* < 0.05; ** *p* < 0.01).

Gene expression was monitored in *A. flavus* AF3357, in the mutant strains *Zn_2_Cys_6_-OE-GFP* and in AFC-1, starting at 14 h through to 5 dai (Figure 3). Time intervals were chosen based on the effects of the fungal invasion observed during the histological analysis (Figure 1). In AF3357, *SalOH* is expressed just 14 h after inoculation as a sign of early infection and it follow a constant trend until 5 dai. The same expression pattern was observed for *npp1* and *AFLA_096260*, from 1 to 4 dai. In strain *Zn_2_Cys_6_-OE-GFP*, *SalOH* had the clearest pattern, that is a constant overexpression as possibly expected. The other two genes instead present a more erratic, even if overxpresssed for some time points, trend; this suggests that other means of controlling the expression of these genes could occur. In AFC-1, *SalOH* and *NPP1* expression levels reflect the fact that this strain is less virulent, as indicated also in Figure 1. Expression levels of the three genes in each strain were as follows: *Zn_2_Cys_6_-OE-GFP* > AF3357 > AFC-1, as shown in Figure 3. Notably, the three genes appeared to be equally important in supporting the pathogenic behaviour of *A. flavus* in maize kernels.

### 2.4. SA and Cathecol Accumulation in Maize-Infected Kernel

**Figure 4:**
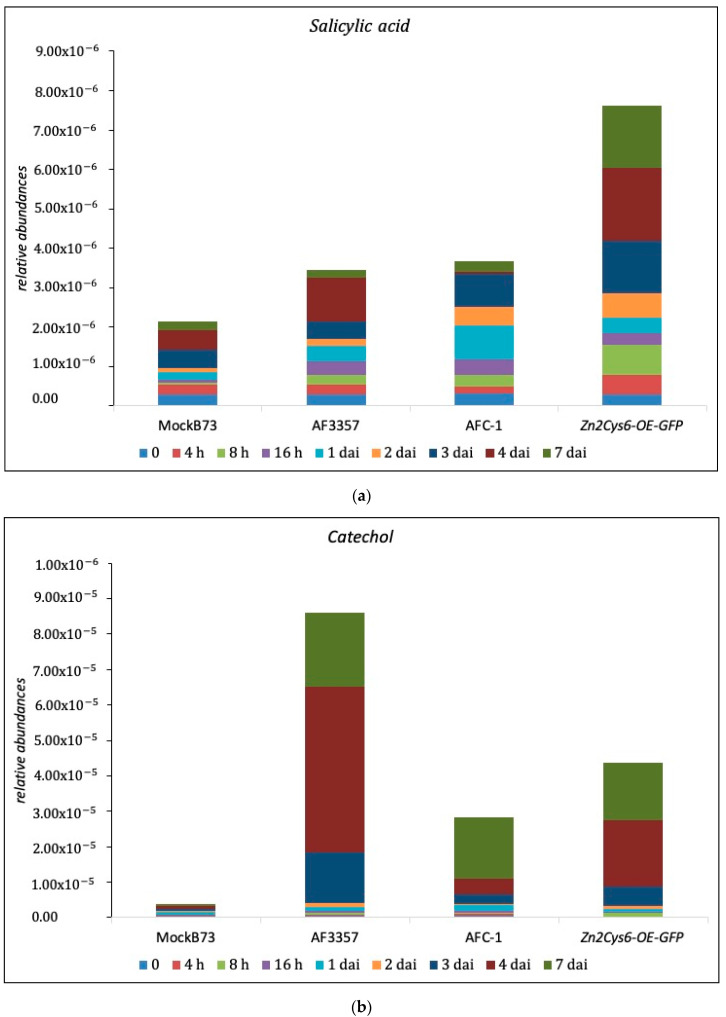
Relative abundances of salicylic acid (**a**) and catechol (**b**) detected in maize when not infected (MockB73) or infected with one of three *A. flavus* strains. The colours in each bar represent different infection time points.

With the aim to verify the activity of fungal *SalOH* into maize kernels infected with several strains (AF3357, AFC-1, *Zn_2_Cys_6_-OE-GFP*), HPLC-MS/MS quantification of both salicylic acid and its by-product catechol was performed. As shown in Figure 4, the progression of SA and its conversion into catechol is related to fungal infection, particularly in the presence of AF3357, presumably in correlation to the expression of *SalOH*. An unexpected difference emerged among the strains: AF3357, the most aggressive in this comparison, is the better converter of SA into catechol (Figure 4). Relating this to the histological and gene expression assays, the ability to convert SA into catechol follows a similar path for the three fungal strains examined (AF3357 > *Zn_2_Cys_6_-OE-GFP* > AFC-1). Nevertheless, the overall amount of conversion is up to 10–15% of the total SA produced by the kernels. The decreasing levels of SA are probably not sufficient to switch kernels’ defences off; instead, the lower percentage of SA should be consistent with a slower reaction to *A. flavus* invasion. Since the resistance of maize to AF is essentially based on a multi-gene horizontal defence [35], slowing the reaction down could cause more damage than expected. In fact, both the infection with AF3357 and with *Zn_2_Cys_6_-OE-GFP* prevented the kernel from germinating (as shown below). In fact, as reported by Nancy Keller [36] more than 25 years ago, following embryo germination, mycelial activity around the embryo ceases, probably as a consequence of antifungal compounds secretion. In our case, the pathogen succeeded in engaging in the invasion of the endosperm even by preventing embryo germination [37].

Also in this study, we tried to report the activity of quercetin dioxygenase encoded by the AFLA_096260 gene. Quercetin dioxygenase converts quercetin, itself a by-product of rutin, into protocatechuoyl-phloroglucinolcarboxylic acid (PPA). Our HPLC-MS/MS analysis was unable to identify this product in the maize kernels from each assayed treatment, even though the in vitro assay proved that AF3357 is able to convert rutin into quercetin [34], and quercetin into PPA (Table S5). It is probable that, even if this activity is important in maize kernel invasion, as indicated by gene expression results (Figure 3), the amount of PPA is below our instruments sensitivity threshold.

### 2.5. Npp1 Induces Necrotic Cell Death 

To highlight the importance of *npp1* in maize kernel invasion by *A. flavus*, two strains presenting the GUS reporter gene have been tested using a histochemical approach: *nepA∆-GUS B9-5* (*npp1* knockout mutant) and *nepA-OE-GUS B5-12* (*npp1* overexpression mutant). In relation to previous results on both gene expression and hormonal analysis, we hypothesize that *A. flavus* biotrophically colonises the kernels in the first stage (0–2 dai) and switches to necrotrophy at a later stage (3–7 dai). In support of this hypothesis, we observed an early expression (Figure 3) and enzymatic conversion of SA (Figure 4) related to *SalOH* activity. Thus, we should expect that the lack of expression of a necrosis factor such as *npp1*, or its overexpression, respectively lead to an anticipation or a delay of kernels colonisation. In fact, Npp1 is uninvolved in the biotrophic stage and acts to prompt the necrotrophic stage, when typical ear rot symptoms appear. 

**Figure 5:**
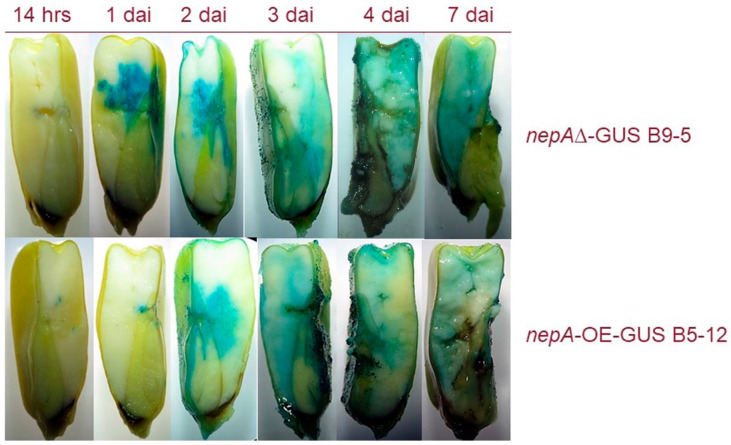
Histochemical assay for *nepA∆-GUS B9-5* and for *nepA-OE-GUS B5-12* strains. Timeline follows infection process starting from the pericarp and the endosperm to the embryo region.

Figure 5 shows the *nepA∆-GUS B9-5* strain invading maize kernels or prior to the *nepA-OE-GUS B5-12* strain (check images at 2 dai for comparison). According to this interpretation, the lack of *npp1* should limit the ability of the *nepA∆-GUS B9-5* strain to colonise the kernels in the later stages (i.e., after 3–4 dai). In fact, the kernels remain viable (radicle emersion at 7 dai) in comparison to the kernels infected with the *nepA-OE-GUS B5-12* strain that show clear symptoms of rot at 3–4 dai.

## 3. Materials and methods

### 3.1. Aspergillus flavus Strains

The *A. flavus* used in this study is the aflatoxin-producer wild-type strain AF3357 [38] and five mutants (Table 1): (i) AFC-1 is a double auxotroph mutant lacking *pyrG* and *argD* gene thus requires arginine and uracil to be supplemented in growth media [16]. Mutant strains (ii) *Zn_2_Cys_6_Δ* and (iii) *Zn_2_Cys_6_Δ-OE-GFP* were generated during this study: both derived from AFC-1 and are auxotrophic for uracil but in the *Zn_2_Cys_6_Δ* strain the transcription factor *Zn_2_Cys_6_* is deleted while in the *Zn_2_Cys_6_Δ-OE-GFP* strain the same gene is instead over-expressed (protocol in Section 3.2 below). Concerning the other two mutants, one strain presents the (iv) *nepA* gene deletion (*nepAΔ-GUS B9-5*) while, in the other (v) *nepA* is overexpressed (*nepA-OE-GUS B5-12;* Gary Payne, not published). The strains first had to be cultured on media (30 °C for 7 days) to create spore suspensions that would be used to inoculate maize kernels. Most of the strains were cultured on Czapek Dox Broth or Agar (Difco), with the mutant strains requiring amendments to the medium of Uracil (*Zn_2_Cys_6_Δ* and *Zn_2_Cys_6_Δ-OE-GFP*) or Uracil and Arginine (AFC-1). The *nepA* mutants were grown on potato dextrose agar medium. All spore suspensions were diluted to a concentration of 1 × 10^6^ spores/mL in sterile distilled water amended with Triton X-100 (0.01%). All the strains, except for the *Zn_2_Cys_6_∆* and *Zn_2_Cys_6_-OE-GFP* mutants, were kindly provided by the laboratory of Professor G.A. Payne (CIFR, North Carolina State University, Raleigh, NC, USA). To facilitate infection, kernels were inoculated with a pin bar wetted with a solution of fungi spores (1 × 10^6^ spores/mL) incubated at 30 °C for various time points and collected based on the protocol used [38]. For each strain, six kernels were inoculated as biological replicates and experiments were at least conducted twice.

### 3.2. Generation of Zn_2_Cys_6_∆ and Zn_2_Cys_6_-OE-GFP Strains

Gene deletion strategies of the transcription factor, *Zn_2_Cys_6_*, were performed through the restoration of auxotrophy in the AFC-1 strain. The *Zn_2_Cys_6_∆* deletion construct was assembled combining three fragments: ~1.5 kb regions upstream (5′ UTR, 1542 bp) and downstream (3′ UTR, 1475 bp) of the *Zn_2_Cys_6_* coding sequence were used as promoter and terminator to regulate the expression of the *argD* marker gene (2225 bp), which encodes for the acetyl ornithine aminotransferase. Each fragment was flanked with specific restriction sites suitably designed adjacent to the primers and added to the amplicons after PCR amplification (Table S6). The restriction enzymes used for cloning [AscI, FesI, Sbf-HF, PacI (NEB, Ipswich, MA, USA)] belong to the family of eight-base cutters whose special feature is the recognition of 8 bp restriction sequences that rarely occur in a genome, which significantly increases their specificity. Fragments were individually cloned into TOPO vector pCR 2.1 (TOPO^®^ CLONING KIT, Invitrogen, Carlsberg, CA, USA) and then used to transform One Shot^®^ Top10 competent cells. This procedure was performed according to the manufacturer’s instruction. Plasmids were extracted using a MINI PREP KIT (Merck KGaA, Darmstadt, Germany) and checked through PCR (Table S6) to assess for the correct insertions. The assembly of the construct started with the double digestion of the vectors harbouring *argD* and 5′ UTR using enzymes AscI and FseI; the reactions occurred over night at 37 °C. After, the excised 5′ UTR fragment and the linearised plasmid containing *argD* were separated by gel electrophoresis, purified using GenElute Gel Extraction Kit (Merck KGaA, Darmstadt, Germany) and quantified. Suitable amounts of the 5′ UTR fragment and the linearized plasmid harbouring *argD* gene were incubated over night at RT with T4 DNA Ligase (Promega, Madison, WIS, USA) with the aim of obtaining a single vector with 5′ UTR fused to the marker. This plasmid was then used to transform Top10 cells by thermal shock and 10 colonies, randomly selected on Luria-Bertani (LB) supplemented with 100 µg/mL of ampicillin, were grown for plasmid recovery. The correct assembly was checked by PCR (Table S6). A similar methodology was adopted for the second part of the assembly in which the fusion of 3′ UTR and 5′ UTR+*argD* was determined. 3′ UTR and 5′ UTR+*argD* were individually double-digested using restriction enzymes SbfI-HF and PacI (NEB, Ipswich, MA, USA). The excised 3′ UTR fragment and the linearised plasmid containing 5′ UTR + *argD* were separated by gel electrophoresis, purified and quantified. The vector harbouring 5′UTR + *argD* was then incubated (overnight at RT) with linear 3′ UTR and T4 DNA Ligase (Promega, Madison, WIS, USA). Thus, the obtained plasmid was used to transform Top10 cells and plated onto LB + Amp 100 µg/mL. Randomly selected colonies were grown over night in LB medium for plasmid recovery. Digestion using EcoRI and PCR amplification were performed to assess the correct assembly of the entire gene-deletion construct. 

The *Zn_2_Cys_6_*-*OE-GFP* strain was obtained by transformation of the auxotrophic mutant AFC-1 strain (*-pyrG; -argD*). The *Zn_2_Cys_6_-OE-GFP* construct was generated by cloning *Zn_2_Cys_6_* gene, placed under the control of the cytomegalovirus (CMV) promoter and fused to green fluorescent protein (GFP), into the pNuc’EM2 plasmid [40]. *Zn_2_Cys_6_* was PCR amplified from *A. flavus* NRRL genomic DNA (primers reported in Table S6) to which sequences containing restriction sites recognized by HindIII and KpnI enzymes (NEB, Ipswich, MA, USA) were added. The PCR-amplified fragment was purified, cloned into plasmid TOPO vector pCR 2.1 with TOPO^®^ CLONING KIT (Invitrogen, Carlsberg, CA, USA) and used to transform One Shot^®^ Top10 chemically competent *E. coli*. The correct insertion of the gene was checked by PCR after plasmid recovery (Table S6). With the aim to excise the *Zn_2_Cys_6_* fragment from pCR2.1 vector and to clone into pNuc’EM2, two individual double digestions of pCR 2.1+*Zn_2_Cys_6_* and pNuc’Em2 plasmid were performed using HindIII and KpnI enzymes (37 °C for 1.5 h). The excised *Zn_2_Cys_6_* and the linearised pNuc’EM2 vector were gel purified with a GenElute Gel Extraction Kit (Sigma, St. Louis, MO, USA). After a ligation step (T4 DNA Ligase, Promega, Madison, WIS, USA), the desired plasmid was used to transform competent Top10 cells and was recovered with a GenElute Plasmid Miniprep Kit (Sigma, St. Louis, MO, USA). Primers CMV_Fw/GFP_Rev were used to test the correct assembly of the CMV-*Zn_2_Cys_6_*-GFP construct (Table S6). Transformation of AFC-1 strain was performed with the aim to obtain all the desired mutants: the linearised plasmid harbouring 5′ UTR-*argD*-3′ UTR was used for the deletion mutant *Zn_2_Cys_6_∆*, while the overexpression construct *Zn_2_Cys_6_*-OE-GFP and the *argD* gene were used to co-transform the AFC-1 strain for the overexpression mutant. All the transformations were performed as reported by He et al. [41]. Transformants were selected on Czapek Dox Agar media plus Uracil after 2–4 days of incubation at 37 °C. 

### 3.3. Zea Mays Crops

The kernels used was Pioneer P1543 FAO class 600 from 130 days, kindly provided by Agricola 2000 srl (Milano, Italy). Maize kernels were surface sterilised with two 5-min washes of 80% ethanol, followed by two 15-min washes with 50% bleach and five rinses with sterile distilled water. Sterilisation was considered necessary to remove environmental residues that could have altered the experiment. 

### 3.4. Gene Expression Analysis 

Total RNA was extracted using a Trizol (Invitrogen, Carlsbad, CA, USA) based protocol with only sterilised material with Diethyl pyrocarbonate (DEPC). RNA was quantified with Qubit RNA Assay kit (Life Technologies, Carlsbad, CA, USA) or Nanodrop (Thermo Scientific, Waltham, MA, USA). To remove DNA contamination, RNA was digested with DNAseI (Promega, Madison, WIS, USA) and complementary DNA (cDNA) was obtained by retro-transcription of 1 µg of RNA using SensyFAST cDNA synthesis kit (Bioline, London, UK). The cDNA was subjected to quantitative analysis of the expression profile for each gene of interest: *SalOH* (salicylate hydroxylase), *npp1* (necrosis- and ethylene-inducing peptide) and AFLA_096026 (aromatic, ring-opening dioxygenase family protein). Gene expression in our *A. flavus* strains was compared to a housekeeping gene (β-tub, AFLA_068620) using a qRT-PCR (see Table S7 for primers). Three technical replicates were used for RT-qPCR.

### 3.5. Rutin Degradation

With the aim to assess rutin degradation, *A. flavus* strain AF3357 was maintained on Czapek Dox Agar, amended with ZnSO_4_ (5 mg/L) and NaMoO_4_ (1 mg/L) at 30 °C. Seven days later the spores were suspended in 5-6 mL of sterile distilled water and Triton X-100 (0.01%, Sigma St. Louis, MO, USA) and inoculated into 100 mL of Potato Dextrose Broth (1 × 10^5^ spores/mL) and grown at 28 °C for 7 days in static. Concomitantly, AF3357 was used to inoculate 100 mL of Potato Dextrose Broth containing rutin 0.02 mg/mL [34]. Culture filtrates were sampled at seven different time intervals from 0 up to 168 h post inoculation (hpi). Then, 2 mL of ethyl acetate were added to 2 mL of culture filtrate add the internal reference standard (chlorogenic acid, SIGMA, St. Louis, MO, USA) at a final concentration of 10 µM. The samples were evaporated to dryness, re-suspended in 200 µL of methanol and finally filtered with a Millex-HV 0.45 µm filter from Millipore (Merck Millipore, Bedford, CA, USA) before HPLC-MS/MS analysis reported below. 

### 3.6. Salicylic Acid, Catechol, Rutin, Quercetin and Aflatoxin B1 Analysis by HPLC-MS/MS

Maize kernels were infected with AF3357, AFC-1, *Zn_2_Cys_6_-OE-GFP* to compare the relative quantity of SA and its by-product catechol. Infected and not infected kernel were freeze-dried and grounded in presence of liquid nitrogen. The extraction and the analysis were performed following the hormone method extraction reported in Scala et al. [42] with some modification. Thirty mg were extracted with 750 µL MeOH–H_2_O–HOAc (90:9:1, *v*/*v*/*v*) in presence of the internal standard 1-Naphthaleneacetic acid (NAA MW 186.21 g·mol^−1^) at 5 μM final concentration. Extraction was repeated and the supernatant was collected and dried by nitrogen flow. Extract was resuspended in 200 µL of 0.05% HOAc in H_2_O–MeCN (85:15, *v*/*v*). Analysis was conducted by HPLC-MS/MS Agilent 6420 (Agilent Technologies, Santa Clara, CA, USA). Chromatographic separation was performed with a Zorbax ECLIPSE XDB-C18 rapid resolution HT 4.6 × 50 mm 1.8 μm p.s. column (Agilent Technologies, Santa Clara, CA, USA) at room temperature, and the injected volume was 10 µL.

The acquisition was in MRM negative ion mode [M − H]^−^ for SA and cathecol. The mobile phases consisted of A: H_2_O containing 0.05% HOAc, and B: Acetonitrile at a constant flow-rate of 600 mL·min^−1^. The elution gradient was as follows: 0–3 min 15% B, 3–5 min 100% B, 5–6 min 100% B, 6–7 min 15% B, 7–8 min 15% B. The gradient was followed by a 5 min re-equilibration. For aflatoxin B1, the acquisition was in MRM positive ion mode [M + H]^+^. Chromatographic separation was performed with a Zorbax Eclipse XDB-C18, 50 × 4.6 mm inner diameter, 1.8 µm particle size, (Agilent Technologies, Santa Clara, CA, USA) at 25 °C and the injection volume was 5 µL. The mobile phases consisted of A: methanol/water/acetic acid 10:89:1 (*v*/*v*/*v*) and B: methanol/water/acetic acid 97:2:1 (*v*/*v*/*v*) while both contained 5 mM ammonium acetate. The total runtime was 20 min, with a flow rate of 0.4 mL/min. The gradient elution was as follows: 0–2 min 1% B, 3–14 min 99% B, 15–18 min 99% B, 19–20 min 1% B. The gradient was followed by 4 min for re-equilibration. 

For rutin and quercetin, the acquisition was in MRM positive ion mode [M-H]^+^. The elution gradient was carried out with binary solvent system consisting of 5 mM NH_4_AcO in H_2_O (solvent A) and 5 mM NH_4_AcO in MeOH (solvent B) at a constant flow-rate of 600 mL·min^−1^. The elution gradient was as follows: 0–2 min 0% B, 0–14 min 100% B, 14–18 min 100% B, with 2 min for re-equilibration. The MRM fragmentation patterns for each compound are listed in Table 2. The values for rutin and quercetin provided as relative abundance are reported in Table S5.

### 3.7. Histological Protocol

Maize kernels (infected and non-infected) were immersed in 70% ethanol for two hours, then 90% ethanol for two hours and next 100% ethanol overnight. Two hours in resin (Technovit^®^ 7100- Haraeus-Kulzer, Wehrheim, Germany)/absolute ethanol (1/3), two hours in resin/absolute ethanol (1/1), and two hours in resin/ absolute ethanol (3/1). Overnight impregnation (resin plus catalyst 1) was performed at 4 °C and then replaced with resin plus catalyst 2 (20:1) at room temperature for resin inclusion. The included samples were then cut with an automatic microtome HM 350 (Zeiss, Oberkochen, Germany) to 8 mm as thickness and stained with toluidine blue for 2 min.

### 3.8. Histochemical Assay

Maize kernels (infected and non-infected) were transferred in cooled (−20 °C) 80% acetone (dilution with distilled water) and chilled for 20 min at −20 °C. Acetone was removed and samples were washed three times with distilled water before the addition of GUS buffer to the samples. An infiltration of 15 min under vacuum was performed, followed by incubation at 37 °C in darkness for at least 2 h. To fix the reaction, the GUS buffer was removed, then replaced with 70% ethanol and preserved at 4 °C. The time points of withdrawals were 4 h, 8 h, 16 h, 24 h, 48 h, 72 h, 96 h and 7 d. No histochemical data is available for the 4 and 8 h time points because *A. flavus* growth was not detectable. The microscopic observation was carried out with objective 40×.

### 3.9. TUNEL Assay

Programmed cell death was evaluated using terminal deoxynucleotidyl transferase (TdT)-mediated dUTP nick-end labelling (TUNEL assay) using a TACS XL™ detection kit (R&D Systems, Minneapolis, MN, USA). Procedures were performed according to the manufacturer’s instructions on kernel sections (6 µm thick) immediately observed under a light microscope (Leica DMRB), and photographs were captured using a by DC500 (Leica, Wetzlar, Germany). Both positive and negative controls were performed according to the manufacturer. The microscopic observation was carried out with objective 100×.

## 4. Conclusions

In general, the biosynthesis of secondary metabolites has not been studied to the same extent of that of primary metabolites. Nevertheless, due to the economic importance of many secondary metabolites, they have more and more been the focus of many research efforts [43,44,45]. The aflatoxin biosynthetic pathway has been well-characterized by several works [5]; yet, information about the regulatory mechanisms is still partial [38,39]. A previous transcriptional analysis on 56 individual secondary metabolite clusters within the genome of *A. flavus* [17] showed a significative patterns of up-regulation, during maize kernel infection. Within this pattern, cluster 32 was especially interesting because it includes two genes involved in the synthesis of Aflatrem, a potent tremorgenic mycotoxin linked to neurological disorders [18]. The objective of the present work was to characterize the role of the genes within cluster 32 which are involved in the early stages of the infection process. Aflatoxin B1 synthesis is not apparently affected by deletion or overexpression of the transcription factor putatively controlling this cluster, even if an intriguingly—not statistically significant—downregulation emerged in the *Zn_2_Cys_6_*∆ strain (compared to AF3357; T test *p* = 0.09) (Table S8). During the first phase of infection (from 2 and 4 dai), we monitored *A. flavus* colonisation of maize kernels by observing histological sections with an optical microscope. As shown in Figure 1, the pathogenic process starts from the external layers and progresses through the aleuronic layer towards the embryo. Our results suggest that *A. flavus* mainly overcomes plant defences in the aleuronic layer to induce PCD in the kernel. This hypothesis is validated by the TUNEL assay, where the presence of apoptotic nuclei becomes evident (Figure 2). In our experimental design, we also included *A. flavus* mutant strains (Table 1, Section 3): data show that AFC-1, auxotroph for arginine and uracil, has a delayed infection onset, while *Zn_2_Cys_6_-OE-GFP*, overexpressing cluster 32, performs similarly to *A. flavus* 3357. To better characterize cluster 32 centrality to the infection process, we looked at the transcriptional levels of three genes within cluster 32: salicylate hydroxylase (*SalOH*), the necrosis and ethylene inducing protein (*npp1*), and quercetin dehydrogenase (AFLA_096260). The elected genes are shown to enable infection by preventing effective plant defence (Figure 3). *Zn_2_Cys_6_-OE-GFP*, the strain overexpressing the transcription factor *Zn_2_Cys_6_*, shows related transcription levels higher than AF 3357 which correlate to a heightened virulence, arguably due to cluster 32 upregulation.

The activity of *SalOH* (converting the salicylic acid in cathecol) was validated also by HPLC/MS-MS. We observed an increased level of cathecol in comparison to the starting level of salicylic acid in all *A. flavus* strains (Figure 4). This evidence corroborates the centrality of cluster 32 in the infection process, since *A. flavus* is able to bypass the plant defences, namely those regulated by SA, by taking advantage of the activity of genes specifically encoded by cluster 32. NLPs are non-specific toxins capable of inducing host tissue necrosis and defence responses [30]. We provide evidence in this sense by showing that *npp1* is involved in *A. flavus* virulence on maize kernels. Experiments on two strains, *nepA-OE-GUS B5-12* and *nepA∆-GUS B9-5*, allowed us to show induced necrotrophy through histochemical assays (Figure 5). The infection starts from the aleuronic layer, which represents the barrier between the plant and the fungus. Maize colonisation by *nepA∆-GUS B9-5* is similar to colonisation by *nepA-OE-GUS B5-12*, which supports the idea that reduced pathogenicity of the *nepA∆-GUS B9-5* strain is not the direct consequence of a decreased growth rate, and suggest a central role of *npp1* in the inter process that sees the saprophytic lifestyle of *A. flavus*.

In *A. flavus*-maize competition, cluster 32 appears to represent the “winning hand” of the pathogen over the host and, by extension, the genetic element that enables the lifestyle switch from biotrophic to necrotrophic. We can thus argue that cluster 32 has acquired a centrality in *A. flavus* ecology, which makes it an interesting topic in future research efforts, especially those aimed at providing the host plant with genetic elements to confer innate resistance.

## Figures and Tables

**Table 1:** *A. flavus* strains.

Strain	Growth Medium	Characteristics	References
***Aspergillus flavus* 3357**	Czapek Dox Broth or Agar (CD, Difco)	Wild type	Payne et al. (2007) NRRL [39]
**AFC-1**	Czapek Dox Broth or Agar (CD, Difco) with Uracil 1.122 g/L and 0.26 g/L of Arginine	Mutant (*−pyrG*, *−argD*) requires uracil and arginine	Georgianna et al. (2010) [16]
***Zn_2_Cys_6_∆***	Czapek Dox Broth or Agar (CD, Difco) with Uracil 1.122 g/L	auxotroph for uracil in which the transcription factor *Zn_2_Cys_6_* has been deleted; it derives from AFC-1 strain with arginine auxotrophy restored	This study
***Zn_2_Cys_6_-OE-GFP***	Czapek Dox Broth or Agar (CD, Difco) with Uracil 1.122 g/L	auxotroph for uracil in which the transcription factor *Zn_2_Cys_6_* is overexpressed; it derives from AFC-1 strain with arginine auxotrophy restored	This study
***nepA-OE-GUS B5-12***	Potato Dextrose Agar (PDA, Difco)	*nepA* gene over-expressing strain	G.A. Payne (not published)
***nepA∆-GUS B9-5***	Potato Dextrose Agar (PDA, Difco)	*nepA* gene knock-out strain	G.A. Payne (not published)

**Table 2:** MRM parameters.

Compound	Precursor Ion	Product Ion	Fragmentor (V)	CE (eV)	Polarity
SA	137.2	92.9, 64.8	135	20	[M-H]^−^
Catechol	109.1	53.1	135	20	[M-H]^−^
NAA	245	180.8	100	16	[M-H]^−^
Quercetin	303.1	152.9	100	37	[M-H]^+^
PPA	291.3	129.1, 112.8	90	38	[M-H]^+^
Chlorogenic acid	353.31	191.2	135	10	[M-H]^−^
Rutin	609.2	273.1	100	56	[M-H]^−^
Aflatoxin B1	313.1	241.1	135	38	[M-H]^+^

## Supplementary Materials

Supplementary materials can be found at https://www.mdpi.com/1422-0067/21/21/8213/s1.

## Abbreviations

AF	Aflatoxin
dai	Day After Inoculation
EFSA	European Food Safety Authority
GUS	β-Glucuronidase
HR	Hypersensitive Response
PCD	Programmed Cell Death
PPA	protocatechuoyl-phloroglucinolcarboxylic acid
ROS	Reactive Oxygen Species
SA	Salicylic Acid
SM	Secondary Metabolites
SMURF	Secondary Metabolite Unknown Regions Finder
TUNEL	Terminal Deoxynucleotidyl Transferase dUTP Nick end Labeling
