# Supplementary material for: Aspergillus flavus Exploits Maize Kernels Using an “Orphan” Secondary Metabolite Cluster
Source: Int J Mol Sci. 2020 Nov 3;21(21):8213. doi: 10.3390/ijms21218213 (PMC7663156; doi:10.3390/ijms21218213)
Supplement: Supplementary file 1 [file ijms-21-08213-s001.pdf]

SUPPLEMENTARY DATA

Supplementary Figure S1. *Aspergillus flavus* plates: a) AF3357, b) AFC-1, c) Zn_2Cys_6-*OE-GFP* and d) $Zn_2Cys_6\Delta$ at 7 days incubation on PDA

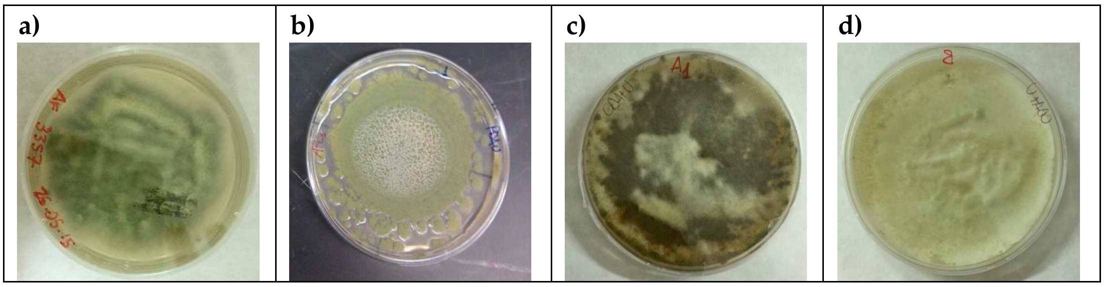

Supplementary Figure S2. *Aspergillus flavus* kernel assay: a) mock, b) AF3357, c) AFC-1, d) Zn_2Cys_6-*OE-GFP* and e) $Zn_2Cys_6\Delta$ at 7 days after inoculation on sterilized maize

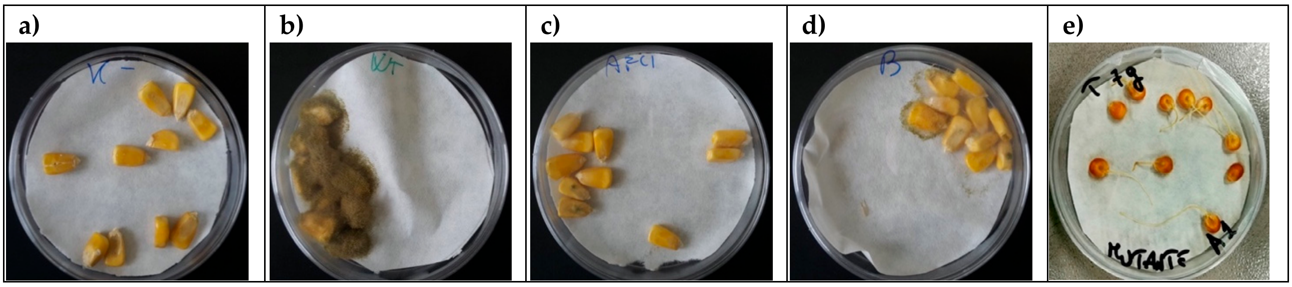

Supplementary Figure S3. Histological assay for mock-inoculated kernel (a) as well as kernel inoculated with *Zn2Cys6Δ* mutant at 2 dai (b) and 7 dai (c) without any apparent sign of infection.

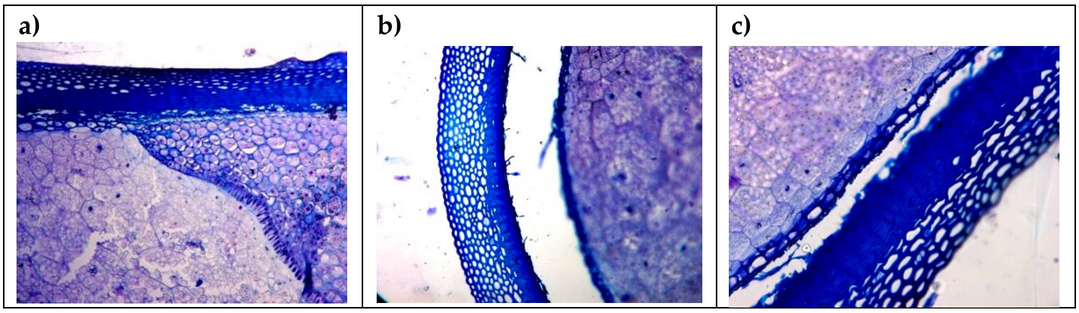

Supplementary Figure S4. TUNEL assay for mock-inoculated kernel (a) as well as kernel inoculated with *Zn2Cys6Δ* mutant at 2 dai (b) where it seems more pathogenic than its control (AFC-1) since the aleuronic layer is detached from the endosperm (a sign of plant stress). At 7 dai, the maize kernel did not show intercellular infection suggesting a slow infection process.

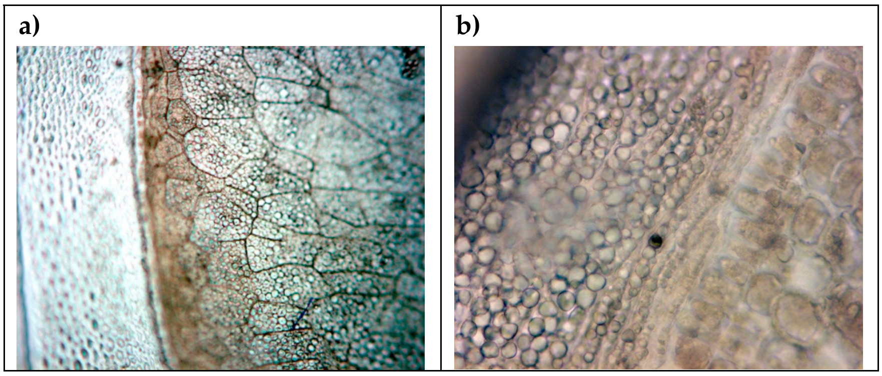

Supplementary Table S5. *A. flavus* can produce enzyme able to hydrolyze rutin through a putative presence of rutin-degrading enzyme (rutin oxidase) into quercetin. Then, the quercetin is degraded by the action of quercetin dehydrogenase (que dox) into 2-protocatechuoylphioroglucinol carboxylic acid. *A. flavus* 3357 grown in presence of rutin was analyzed by HPLC-MS/MS at 0 and 7 days after inoculation (dai). Rutin, quercetin and 2-protocatechuoylphioroglucinol carboxylic were detected.

	rutin	quercetin	2-protocatechuoylphioroglucinol carboxylic acid.
Relative abundance at 0 dai	44593,79 ± 2658,25	<LOD	<LOD
Relative abundance at 7 dai	3768,30 ±568,22	7628,50 ± 526,33	3625,45 ± 458,32

Supplementary Table S6. Primers used to generate the mutant strains.

**Primer name**	**Primer sequence 5′-3′**	**Region amplified and its size**	**Aim of the set of primers**
5 U1	TCAGTGAGGCTCGTGGTTTT	5 UTR 1558 amplicon size	To assembly the three fragments for AFLA_096370 (Zn_2Cys_6) deletion
5 U2	AGATTTACGCCTTGGCTCCT		
Arg FW	AATTGCGGAGCAAATCACA	argD 2356 amplicon size	
Arg Rev	TTCCTCTGATGCAGGGATTC		
3 U1	GACTAAGCCTCGCCCATTTG	3 UTR 1491 amplicon size	
3 U2	TTGATCAGGCAAGGTCCTCG		
465 Fw	CACCCGGATATCCTGACCTC	2515 amplicon size	PCR to confirm the correct transformation in $Zn_2Cys_6\Delta$ mutant
3U4	TTAATTAATTGATCAGGCAAGGTCCTCG		
465 Fw	CACCCGGATATCCTGACCTC	2515 amplicon size	
AfZn_P6	TTGATCAGGCAAGGTCCTCG		
465 Fw	CACCCGGATATCCTGACCTC	2484 amplicon size	
AfZn_P10	CCATTCGTCCACCATTGCAT		
ZnFW1	GGGCCGAATCTTAATCGTCG	2771 amplicon size	Zn_2Cys_6 for overexpression construct
ZnRev2	ACATGAACACTGGGTAGCGA		

AF_534 Fw	GCGATGCAGAGAGAAGAAGC	*Zn_2Cys_6*<br>534 amplicon size	Southern blotting probe
AF_534 Rev	AGTATACGCCGAGTCAAGGG		

Supplementary Table S7. RT-qPCR primers.

Gene	Sequences	Base pair
Afbtub_fw (AFLA_068620)	GTGACCACCTGTCTCCGTTT	211 amplicon size
Afbtub_rev (AFLA_068620)	GGAAGTCAGAAGCAGCCATC	
SalOHfw (AFLA_057780)	TCGCTCCTCTCATCCATCAC	133 amplicon size
SalOHrev (AFLA_057780)	CGGAGCGGCAATTCACTTAA	
NPP1fw (AFLA_096450)	AAGACGAACAACCCACAACG	134 amplicon size
NPP1rev (AFLA_096450)	GGAAGAGACTCCCAGGCAAT	
AF_QD_Fw (AFLA_096026)	CATCCTCCGACTATGCGTTTAC	124 amplicon size
AF_QD_Rev(AFLA_096026)	CTGGAAGCGACCCTTGAAAT	
CMV_Fw	CGCAAATGGGCGGTAGGCGTG	3047 amplicon size
GFP_Rev	AATGGTCTGCTAGTTGAACGC	

Supplementary Table S8. Aflatoxin B1 quantification in the strains of *A. flavus* used in this study. Maize kernels infected with the strain specified in the table were extracted as indicated in the methods section at 7 dai.

Strain	AFB1 quantification (ppb)
a) AF3357	151.3 ± 10.2
b) AFC-1	85.2 ± 6.5
c) *Zn_2Cys_6-OE-GFP*	138 ± 15.6
d) *$Zn_2Cys_6\Delta$*	125 ± 11.8
e) *nepAΔ-GUS B9-5*	141.6 ± 20.5
f) *nepA-OE-GUS B5-12*	184.3 ± 26.2
